# Racial disparities in breast cancer preclinical and clinical models

**DOI:** 10.1186/s13058-022-01551-x

**Published:** 2022-08-05

**Authors:** Shannique Clarke, Sheray N. Chin, Leah Dodds, Sophia H. L. George, Simone Badal

**Affiliations:** 1 Department of Basic Medical Sciences, Faculty of Medical Sciences Teaching and Research Complex, The University of the West Indies, West Indies, Mona Jamaica; 2 Department of Pathology (Division of Haematology and Oncology), Faculty of Medical Sciences, University of the West Indies, Mona, Jamaica; 3 Department of Obstetrics, Gynecology and Reproductive Sciences, Division of Gynecologic Oncology, Sylvester Comprehensive Cancer Center, University of Miami Miller School of Medicine, Miami, FL USA

**Keywords:** Breast cancer, Black, White, Cell lines, Clinical trials, Disparity

## Abstract

Breast cancer (BCa) has long been a health burden to women across the globe. However, the burden is not equally carried across races. Though the manifestation and behavior of BCa differs among racial groups, the racial representation of models used in preclinical trials and clinical trial participants lacks this heterogeneity. Women of African Ancestry (WAA) are disproportionately afflicted by having an increased risk of developing BCas that are more aggressive in nature, and consequently suffer from poorer outcomes relative to women of European ancestry (WEA). Notwithstanding this, one of the most commonly used tools in studying BCa, cell lines, exhibit a sizeable gap in cell line derivatives of WEA relative to WAA. In this review, we summarize the available BCa cell lines grouped by race by major suppliers, American Type Culture Collection (ATCC) and the European Collection of Authenticated Cell Cultures (ECACC). Next, examined the enrollment of WAA in clinical trials for BCa. Of the cell lines found provided by ATCC and ECACC, those derived from WEA constituted approximately 80% and 94%, respectively. The disparity is mirrored in clinical trial enrollment where, on average, WEA made up more than 70% of participants in trials found where ancestry information was provided. As both experimental models and clinical trial participants primarily consist of WEA, results may have poorer translatability toward other races. This highlights the need for greater racial diversity at the preclinical and clinical levels to more accurately represent the population and strengthen the translatability of results.

**Supplementary Information:**

The online version contains supplementary material available at 10.1186/s13058-022-01551-x.

## Background

**Fig. 1:**
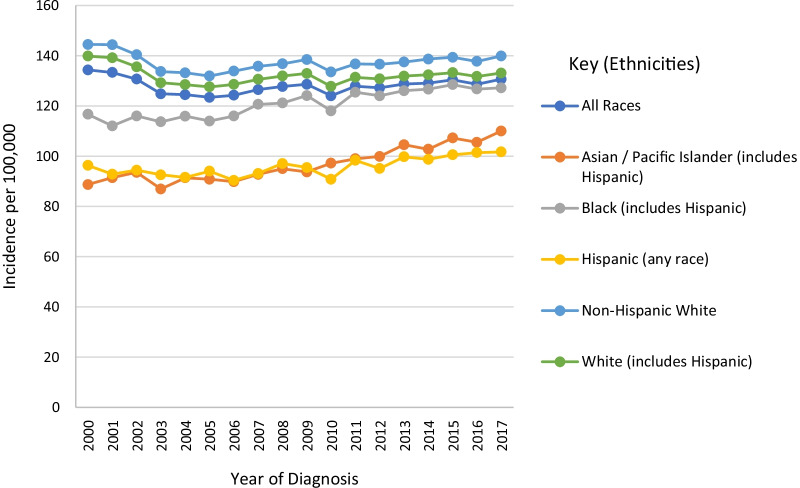
Trends in breast cancer age and delay-adjusted incidence rates for all stages of the four primary molecular subtypes from 2000 to 2017. *Data obtained from SEER 21 registries* [2]

**Fig. 2:**
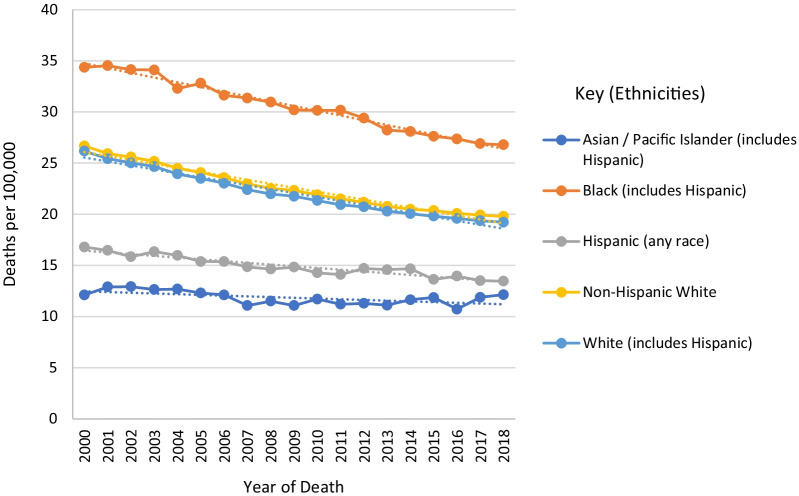
Trends in breast cancer age-adjusted mortality rates for all breast cancers of the four primary molecular subtypes from 2000 to 2018. *Data obtained from SEER with data from the US Mortality Files, National Center for Health Statistics, and CDC* [2]

Women of African ancestry (WAA) are 40% more likely to die of BCa than women of European ancestry (WEA) and have the highest death rates due to BCa of all racial groups [1]. Data from the Surveillance, Epidemiology, and End Results Program (SEER) [2] show a 5-year (2013–2017) age and delay-adjusted incidence of 128.5 per 100,000 women of all races, with a general increase in incidence from 2005 (Fig. 1). Non-Hispanic WEA have a higher than average 5-year incidence of 137.3 per 100, 000 while WAA (including Hispanics) have a below average rate of 124.8 per 100,000. However, despite having a higher incidence, Non-Hispanic WEA have a lower mortality rate of 19.8 per 100, 000 relative to 26.8 per 100, 000 for WAA, who have the highest mortality rate among all races (Fig. 2).

Women of African ancestry are usually diagnosed with BCa at advanced stages and are disproportionally diagnosed with the most aggressive form of BCa [3, 4]. Several factors have been identified as contributors to cancer health disparities. Some examples include: a lack of trust in health systems, access to quality care, increased time to treatment, access to Academic cancer centers, implicit bias, and neighborhood segregation [5–7]. Risk factors associated with the development of BCa linked with race are less defined and poorly understood in the African descendant population. This paucity of information is probably further amplified due to unpredictable admixed genetic contributions [8].

## Methods

Breast cell lines from the American Type Culture Collection (ATCC) and European Collection of Authenticated Cell Cultures (ECACC) databases were found using the search terms: “breast cell lines,” “breast cells,” and “breast cancer cell lines.” Both cancerous and non-cancerous cell lines were included when compiling the list of available cell lines. The race of the individual from which the cell lines were derived was determined based on information provided by ATCC and ECACC. In cases where race was not specified by these suppliers, ancestry information was obtained from the Swiss Institute of Bioinformatics.

### Inclusion criteria

Trials included in Additional file 1: Table 1 were chosen solely based on the availability of racial background information of their participants. Breast cancers from post and premenopausal females of various stages, histological types, and molecular subtypes were considered.

**Table 1:** Racial distribution of ATCC’s breast cell line panel constituents

Panel	N	Cell lines	Race
ATCC breast cancer cell panel	45	184B5	Unspecified
		AU-565	Caucasian
		BT-20	Caucasian
		BT-474	Caucasian
		BT-483	Caucasian
		BT-549	Caucasian
		CAMA-1	Caucasian
		DU4475	Caucasian
		HCC38	Caucasian
		HCC70	African
		HCC202	Caucasian
		HCC1187	Caucasian
		HCC1395	Caucasian
		HCC1419	Hispanic
		HCC1428	Caucasian
		HCC1500	African
		HCC1569	African
		HCC1599	Caucasian
		HCC1806	African
		HCC1937	Caucasian
		HCC1954	East Indian
		HCC2157	African
		HCC2218	Caucasian
		Hs 578Bst	Caucasian
		Hs 578 T	Caucasian
		MCF7	Caucasian
		MCF 10A	Caucasian
		MCF 10F	Unspecified
		MCF-12A	Caucasian
		MDA-kb2	Caucasian
		MDA-MB-134-VI	Caucasian
		MDA-MB-157	African
		MDA-MB-175-VII	African
		MDA-MB-231	Caucasian
		MDA-MB-361	Caucasian
		MDA-MB-415	Caucasian
		MDA-MB-436	Caucasian
		MDA-MB-453	Caucasian
		MDA-MB-468	African
		SK-BR-3	Caucasian
		T47D	Caucasian
		UACC-812	Mixed; Native American
		UACC-893	Caucasian
		ZR-75–1	Caucasian
		ZR-75–30	African
Breast cancer biomarkers cell line panel	7	UACC-812	Mixed; Native American
		UACC-893	Caucasian
		UACC-3199	Caucasian
		UACC-3133	Caucasian
		UACC-1179	Unspecified
		UACC-732	Caucasian
		UACC-2087	Unspecified
Triple-negative breast cancer panel 1; basal-like morphology	9	HCC1599	Caucasian
		HCC1937	Caucasian
		HCC1143	Caucasian
		MDA-MB-468	African
		HCC38	Caucasian
		HCC70	African
		HCC1806	African
		HCC1187	Caucasian
		DU4475	Caucasian
Triple-negative breast cancer panel 2, mesenchymal & luminal morphology	6	BT-549	Caucasian
		Hs 578 T	Caucasian
		MDA-MB-231	Caucasian
		MDA-MB-436	Caucasian
		MDA-MB-157	African
		MDA-MB-453	Caucasian
Triple-negative breast cancer panel 3	17	HCC1599	Caucasian
		HCC1937	Caucasian
		HCC1143	Caucasian
		MDA-MB-468	African
		HCC38	Caucasian
		HCC70	African
		HCC1806	African
		HCC1187	Caucasian
		DU4475	Caucasian
		BT-549	Caucasian
		Hs 578 T	Caucasian
		MDA-MB-231	Caucasian
		MDA-MB-436	Caucasian
		MDA-MB-157	African
		MDA-MB-453	Caucasian
		BT-20	Caucasian
		HCC1395	Caucasian
Breast cancer p53 hotspot mutation cell panel	8	MDA-MB-175-VII	African
		MDA-MB-361	Caucasian
		AU565	Caucasian
		SK-BR-3	Caucasian
		HCC70	African
		BT-549	Caucasian
		HCC38	Caucasian
		MDA-MB-468	African

N = number of cell lines

Data compiled from the ATCC and Swiss Institute of Bioinformatics [14, 15]

*Note.* Caucasian refers to WEA

### Exclusion criteria

With the exception of the antiestrogen effect of IC146474 clinical trial, clinical trials that did not provide the racial background of its participants were excluded. Cases of male breast cancer were not considered.

### Limits

No limitation was placed on the date for studies included in the literature review.

## Preclinical models as instruments for investigating breast cancer biology

The systematic approach utilized over the years to understand and treat cancers includes preclinical models followed by clinical trials. Preclinical models comprise in vitro and in vivo research and, in both instances, the application of 2D models, cell lines that are also used as organoids and 3D cultures are all vital. Taking sociological factors into account, a closer look at the representational BCa models that have been used over the decades to understand, treat, and prevent BCa is crucial. As described above, there is a clear disparity in incidence and mortality rates for WAA with BCa. What is more is that women of WEA have higher incidence rates, yet women of WAA have higher mortality rates and are more prone to developing the more aggressive and least studied form of BCa—triple negative breast cancer (TNBC). This then begs the question, as to how much do socioeconomical factors contribute versus lifestyle and genetic predisposition. As scientists and clinicians, our preclinical models and clinical trials are primarily employed to address and find answers for the latter reasons and so this review aims to take a closer look at the representational models used to understand, treat, and prevent BCas.

Cultured cells have proven to be cornerstone catalysts in propelling oncology by acting as disease models. Such models have allowed for the elucidation of pathways responsible for cancer initiation and progression, the study of the behavior of cancers, and the discovery of novel therapeutic agents. Although primary cells represent the most appropriate models for analysis owing to their close resemblance to the source tissue, their limited lifespans in culture present a roadblock to long-term investigation. Cell lines remedy this by circumventing the major barrier to immortality—senescence.

Although BCa is of a heterogenous nature, the racial diversity of commercially available cell lines borders on the homogeneous. This is reflected in the catalogue of breast cell lines provided by two major suppliers of cell lines: the ATCC and the ECACC. Of ATCC’s breast cell lines where race could be determined (Additional file 2: Table 2), approximately 80% of those found are derivatives of WEA while those derived from WAA only constitute roughly 14%. The gap widens when examining those supplied by the ECACC as 94% of those found with specified races were derived from WEA while WAA made up the remaining 6%. Across both suppliers, breast cell line derivatives of WEA constitute 89% while those of African ancestry account for only 8%. This disparity is not unique to BCa. Prostate cancer (PCa), which is more prevalent among men of African ancestry (MAA) than any other race, has disproportionally more prostate cell lines derived from men of European ancestry supplied by the ATCC, ECACC, and Sigma-Aldrich with 97% of the 32 listed cell lines being of European origin [9].

The lack of racial diversity in the available cell lines consequently translates to the same patterns in cell line panels. These panels allow for a group of cell lines with overlapping biological characteristics to be studied in tandem, for instance when screening novel therapeutic agents against a selected disease [10]. ATCC’s current lineup includes 6 human breast cell line panels (Table 1) where the cell lines from WEA vastly outnumber those from other races with roughly 67% being derived from WEA. Noteworthy is the disproportionate ratio of European descent to African descent cell lines in the TNBC panels, albeit studies have shown that TNBC is more prevalent among WAA, who also typically have a poorer prognosis [11, 12]. A preclinical trial that aimed to target mutated *p53* in patients with TNBC using the drug PK11007 screened a panel of 17 cell lines. Of these, only 1 was found to be a derivative of a WAA, MDA-MB-468, while 15 were derived from WEA [13].

The first BCa cell line in history, BT-20, was established in 1958 from a 74-year-old WEA diagnosed with TNBC [16]. Later in 1973, the most commonly used BCa model, MCF-7, was isolated from a 69-year-old WEA. Its popularity is due in part to the retention of its ER status to serve as a model for luminal invasive BCa, with its most significant contribution being toward the development of targeted therapies for ER positive BCa [17]. Following MCF-7 in popularity are MDA-MB-231 and T-47D [16], both of which were derived from WEA. Consequently, there is an under representation of cell lines derived from non-European descendant donors in preclinical trials [18–22]. While the contributions of these cell lines should not be minimized, as cell lines derived from WEA, and since the manifestation and behavior of diseases vary among different racial groups [23], the results from studies where they act as a cancer model may hold less external validity toward other races.

Although in vitro culture of cell lines is relatively inexpensive and easier to manipulate, the 2D environment, culture media, and lack of the tumor microenvironment affects the behavior of cells. Consequently, results of preliminary assessments where they’re used as models may not be mirrored in clinical settings. An answer to this is creating an in vivo model using the cell lines in severely immunocompromised rats known as cell line xenographs. Although this model provides a microenvironment, it is still handicapped by cell lines’ lack of heterogeneity and deviations from the original tissue [24]. Three-dimensional models such as organoids and patient-derived xenographs offer a step up in mimicking the original characteristics of their tissue of origin [24–26].

## Clinical trials in breast cancer treatment and prevention

Clinical trials are essential in determining how well results from in vitro and preclinical studies translate to the desired therapeutic effects in humans as well as assessing adverse reactions. A study published in 1971 showcased data of one of the early modern day BCa clinical trials where the antiestrogen effect of IC146474 (now known as tamoxifen) was examined. The trial, in which tamoxifen was used to treat hormone sensitive BCa, involved 46 patients (races not specified) for a period of more than 3 months [27]. Later, in 1992, the NSABP conducted the first BCa chemoprevention trial in history where 13,388 women were recruited to assess tamoxifen’s chemopreventive efficacy. Of the women recruited, 12,706 (94.9%) were WEA, 220 (1.6%) were WAA, and other races made up the remainder [28].

The racial disparity in BCa clinical trial enrollment is evident across trials supported by several organizations (Additional file 1: Table 1). *Scientific American* reports that although racial minorities constitute 40% of the US population, they are typically vastly outnumbered in clinical trials by participants of European descent, who can constitute up to 80–90% of recruits [23].

The issue of racial disparity in BCa trial enrollment is especially evident in trials looking specifically at TNBC. Despite being more prevalent in WAA, there is underrepresentation of WAA in trials specifically studying treatment options for patients with this aggressive BCa subtype. Trophoblast cell surface antigen 2 (Trop-2) is expressed across all BCa subtypes; however, overexpression appears more common in aggressive disease subtypes. Trop-2 is expressed in the majority of TNBCs and may be considered an actionable biomarker. Sacituzumab govitecan-hziy is an antibody–drug conjugate that targets Trop-2 for the selective delivery of SN-38. In 2020, Sacituzumab govitecan (Trodelvy; Immunomedics, Inc.) was approved by the FDA for the treatment of patients who have received at least two prior therapies for metastatic disease [39]. The phase 1/2 clinical trial recruited 108 patients, of which 82 (75.9%) were WEA, 8 (7.4%) were WAA, and 3 (2.8%) were Asian [40].

In the IMpassion130 phase 3 clinical trial (NCT02425891, F. Hoffmann-La Roche/Genentech) of Atezolizumab and Nab-Paclitaxel in advanced TNBC, it was shown that the addition of the immunotherapy drug Atezolizumab to chemotherapy (nab-paclitaxel) prolonged progression-free survival among patients with metastatic TNBC. In this TNBC specific study, the study population was 67.5% WEA (609 patients), with WAA comprising 6.5% (59 patients) [32]. The results of the Impassion130 clinical trial led to FDA approval of Atezolizumab and Nab-Paclitaxel for patients with previously untreated metastatic TNBC.

Although the aforementioned clinical trials did not stratify treatment outcome data by race, a study examined 6676 BCa patients (662 were WAA) enrolled in phase 3 clinical trials carried out by the Southwest Oncology Group (SWOG) between 1974 and 2009. When factors such as stage, therapy, and follow-up were kept equal, WAA had poorer survival relative to WEA. The WAA diagnosed with early-stage premenopausal BCa, regardless of receptor status, had increased mortality with a hazard ratio (HR) for death of 1.41, 95% confidence interval (CI) = 1.10—1.82; *P* = 0.007. The association was also seen with early-stage post-menopausal BCa with a HR for death of 1.49, 95% CI = 1.28—1.73; *P* < 0.001 [41]. Overall, WAA only made up 9.92% of patients enrolled in these SWOG BCa clinical trials. Under representation of WAA in clinical trials continues to be of concern, especially for TNBC-specific clinical trials as WAA are known to be disproportionately affected by this disease. On the other hand, a study that examined data from 13 NSABP clinical trials showed that WAA and WEA treated with tamoxifen showed a near equal reduction in contralateral BCa risk. The risk ratio (RR) reported for the studies’ WAA was 0.74, 95% CI = 0.46—1.17 while the WEA had a RR of 0.76, 95% CI = 0.59 to 0.98 [42]. However, as the area of disparities in treatment outcomes between races is lacking, there is consequently not enough information available to draw a decisive conclusion that suggests treatment efficacy is correlated to race.

## Future considerations

The field of medicine is evolving toward a more personalized approach to patient care, aimed at improving outcomes while reducing treatment-related toxicities. This personalized approach has to begin by systematically addressing commonalities and differences in biomarker expression, at the somatic and germline levels among races taking into account the geographical locale and socio-economic factors among women with BCa. Toward this end, an intentional course at increasing the representation of preclinical models and enrollment in clinical trials is necessary. As such, this needs to be factored in at the levels of funding agencies, IRB, suppliers of in vitro models, and sponsors/investigators of clinical trials. For example, the African Genome Registry (AGR) project a part of the African Caribbean Cancer Consortium (AC3) group, to be undertaken by researchers of the African-Caribbean scNetwork aims to address genetic differences between cancerous and neighboring normal cells of prostate, breast, and cervical cancers from MAA and WAA. This would be done via single cell sequencing of the aforementioned cancers from patients living in the USA, Caribbean, and Africa. Similarly, a current clinical trial project, IRONMAN that is amassing the largest registry of men with advanced PCa has been intentional about increasing the representation of MAA with PCa. Another project similar in nature to IRONMAN under the AGR aims to create a registry of patients of African descent with BCa and PCa of all types and stages from the USA, Caribbean, and Africa. These are being done by strategically partnering with various organizations like the African-Caribbean Cancer Consortium (AC3) and Prostate Cancer Transatlantic Consortium (CaPTC), research consortia that include research groups in the Caribbean and Africa that are high in representation from people of African ancestry. Notwithstanding, tackling BCa wholistically, has to also consider socioeconomic factors such as access to healthcare, dietary trends, and environmental factors, which will maximize the improvement of patient care for women with BCa.

## Conclusion

For the efficacy of therapeutic agents and the findings from all stages of research to have stronger external validity, consideration must be taken when choosing in vitro models and recruiting patients such that they reflect that population they intend to benefit. It was demonstrated that a clear racial disparity exists among the most commonly used in vitro models, cell lines, where on average those derived from WEA constituted more than 80% of those commercially available from major suppliers. Consequently, if a conscious effort is not made to include the fewer cell lines from racial minorities, those used as disease models skew toward European derivatives. The same can be said for the lack of diversity in clinical trials, especially those geared toward TNBC, which disproportionately affect WAA. At this stage, it becomes even more critical for racial equality among study participants as these results will determine if therapies advance to the final stages to eventually become available as treatment options to the public. Acknowledging and shedding light on the issue at hand is the first step in moving toward more inclusive research that, over time, ensures that clinical outcomes are more favorable toward racial minorities.

## Supplementary Information

**Additional file 1. **Racial diversity of breast cancer clinical trials funded by various organizations *N* = number of participants.

**Additional file 2. **Cell lines supplied by the ATCC and ECACC disaggregated by race *N* = number of cell lines.

## Data Availability

The datasets used to the generate the graphs are available from the NIH’s SEER application, https://seer.cancer.gov/explorer/application.html?site=55&data_type=1&graph_type=10&compareBy=race&chk_race_1=1&chk_race_4=4&chk_race_3=3&chk_race_6=6&chk_race_8=8&series=9&sex=3&age_range=1&stage=101&advopt_precision=1&advopt_show_ci=on&advopt_display=2. [2]

## Abbreviations

AC3: African-Caribbean cancer consortium
AGR: African genome registry
ATCC: American type culture collection
BCa: Breast cancer
CaPTC: Prostate cancer transatlantic consortium
CI: Confidence interval
ECACC: European collection of authenticated cell cultures
HER2: Human epidermal growth factor receptor 2
HR: Hazard ratio
MAA: Men of African ancestry
NSABP: National surgical adjuvant breast and bowel project
PCa: Prostate cancer
RR: Risk ratio
SEER: Surveillance, epidemiology, and end results program
SWOG: Southwest oncology group
TNBC: Triple negative breast cancer
WAA: Women of African ancestry
WEA: Women of European ancestry
