# Supplementary material for: Racial disparities in breast cancer preclinical and clinical models
Source: Breast Cancer Res. 2022 Aug 5;24:56. doi: 10.1186/s13058-022-01551-x (PMC9354441; doi:10.1186/s13058-022-01551-x)
Supplement: Supplementary file 1 — Additional file 1. Racial diversity of breast cancer clinical trials funded by various organizations N = number of participants. [file 13058_2022_1551_MOESM1_ESM.docx]

**Supplementary Table 2** Racial diversity of breast cancer clinical trials funded by various organizations

N = number of participants

| Therapeutic Agent(s) | NCT# | Funding  Organization(s) | N | Racial  Distribution | Source |
| --- | --- | --- | --- | --- | --- |
| Palbociclib and Letrozole | [NCT01740427](http://clinicaltrials.gov/show/NCT01740427) | Pfizer | 666 | Caucasian – 516 (77.5%)  African – 11 (1.7%)  Asian – 95 (14.3%)  Other – 44 (6.6%) | [29] |
|  |  |  |  |  |  |
| Trastuzumab Emtansine | [NCT00829166](http://clinicaltrials.gov/show/NCT00829166) | F. Hoffmann -  La Roche/Genentech | 991 | Caucasian – 732 (73.9%)  African – 50 (5%)  Asian – 180 (18.2%)  Unknown/Other – 29 (2.9%) | [30] |
|  |  |  |  |  |  |
| Tucatinib, Trastuzumab, and Capecitabine | [NCT02614794](http://clinicaltrials.gov/show/NCT02614794) | Seattle Genetics | 612 | Caucasian – 444 (72.5%)  African – 55 (9%)  Asian – 23 (3.8%)  Unknown/Other – 90 (14.7%) | [31] |
|  |  |  |  |  |  |
| Atezolizumab and Nab-Paclitaxel | [NCT02425891](http://clinicaltrials.gov/show/NCT02425891) | F. Hoffmann -  La Roche/Genentech | 902 | Caucasian – 609 (67.5%)  African – 59 (6.5%)  Asian – 161 (17.8%)  Unknown/Other – 73 (8.1%) | [32] |
|  |  |  |  |  |  |
| Trastuzumab Deruxtecan | [NCT03248492](http://clinicaltrials.gov/show/NCT03248492) | Daiichi Sankyo  and  AstraZeneca | 184 | Caucasian – 101 (54.9%)  Asian – 70 (38%)  Unknown/Other – 13 (7.1%) | [33] |
|  |  |  |  |  |  |
| Palbociclib | [NCT01942135](http://clinicaltrials.gov/show/NCT01942135) | Pfizer | 521 | Caucasian – 385 (73.9%)  Asian – 105 (20.2%)  African /Other – 31 (6%) | [34] |
|  |  |  |  |  |  |
| Pertuzumab, Trastuzumab, and Docetaxel | [NCT00567190](http://clinicaltrials.gov/show/NCT00567190) | F. Hoffmann -  La Roche/Genentech | 808 | Caucasian – 480 (59.4%)  African – 30 (3.7%)  Asian – 261 (32.3%)  Other – 37 (4.6%) | [35] |
|  |  |  |  |  |  |
| Paclitaxel and Trastuzumab | [NCT00542451](http://clinicaltrials.gov/show/NCT00542451) | Genentech | 406 | Caucasian – 351 (86.5%)  African – 28 (6.9%)  Asian – 11 (2.7%)  Other – 16 (3.9%) | [36] |
|  |  |  |  |  |  |
| Bevacizumab | [NCT00408408](http://clinicaltrials.gov/show/NCT00408408) | NCI and others | 1206  (Race  provided for 600) | Caucasian – 500 (83.3%)  African – 80 (13.3%)  Other – 20 (3.3%) | [37] |
|  |  |  |  |  |  |
| Gemcitabine and Carboplatin  with Iniparib | [NCT00540358](http://clinicaltrials.gov/show/NCT00540358) | BiPar Sciences | 123 | Caucasian – 96 (78%)  African – 21 (17%)  Asian – 1 (0.8%)  Unknown – 5 (4.1%) | [38] |

*Note. Caucasian refers to WED*
