# Supplementary material for: Racial disparities in breast cancer preclinical and clinical models
Source: Breast Cancer Res. 2022 Aug 5;24:56. doi: 10.1186/s13058-022-01551-x (PMC9354441; doi:10.1186/s13058-022-01551-x)
Supplement: Supplementary file 2 — Additional file 2. Cell lines supplied by the ATCC and ECACC disaggregated by race N = number of cell lines. [file 13058_2022_1551_MOESM2_ESM.docx]

**Supplementary Table S1** Cell lines supplied by the ATCC and ECACC disaggregated by race

N = number of cell lines

| Name | Supplier | Patient age | Notes |
| --- | --- | --- | --- |
| African, N = 11 | | | |
| MDA-MB-468 | ATCC | 51 |  |
| MDA-MB-175-VII | ATCC | 56 |  |
| ZR-75-30 | ATCC, ECACC | 47 |  |
| MDA-MB-157 | ATCC, ECACC | 44 |  |
| HCC1008 | ATCC | 67 |  |
| HCC1806 | ATCC | 60 |  |
| HCC1500 | ATCC | 32 |  |
| HCC2157 | ATCC | 48 |  |
| HCC70 | ATCC | 49 |  |
| HCC1569 | ATCC | 70 |  |
| MB 157 | ATCC | 48 |  |
| Caucasian, N = 122 | | | |
| Hs 281.T | ATCC | 37 |  |
| Hs 343. T | ATCC | 47 |  |
| Hs 274.T | ATCC | 66 |  |
| Hs 362.T | ATCC | 78 |  |
| MDA-MB-361 | ATCC, ECACC | 40 |  |
| AU565 | ATCC | 43 |  |
| MCF-7 | ATCC | 69 |  |
| MDA-MB-231 | ATCC, ECACC | 51 |  |
| MDA-MB-415 | ATCC | 38 |  |
| MDA-MB-436 | ATCC | 43 |  |
| CAMA-1 | ATCC | 51 |  |
| SK-BR-3 | ATCC | 43 |  |
| UACC-1179 | ATCC | 61 |  |
| UACC-2087 | ATCC | 51 |  |
| Hs 741.T | ATCC | 47 |  |
| Hs 748.T | ATCC | 48 |  |
| Hs 578T | ATCC, ECACC | 74 |  |
| BT-20 | ATCC | 74 |  |
| Hs 579.Mg | ATCC | Unspecified |  |
| BT-483 | ATCC | 23 |  |
| BT-549 | ATCC | 72 |  |
| BT-474 | ATCC | 60 |  |
| ZR-75-1 | ATCC | 63 |  |
| T47D-KBluc | ATCC | 54 | Derived from T-47D |
| T-47D | ATCC | 54 | Derived from T-47 |
| MDA-MB-134-VI | ATCC | 47 |  |
| UACC-2648 | ATCC | 69 |  |
| Hs 564(E).Mg | ATCC | 53 |  |
| UACC-3199 | ATCC | 58 |  |
| Hs 319.T | ATCC | 42 |  |
| UACC-3133 | ATCC | 52 |  |
| UACC-732 | ATCC | 33 |  |
| MDA-MB-453 | ATCC | 48 |  |
| UACC-893 | ATCC | 57 |  |
| Hs 742.T | ATCC | 71 |  |
| HCC1395 | ATCC | 40 |  |
| HCC1143 | ATCC | 52 |  |
| HCC1187 | ATCC | 41 |  |
| HCC38 | ATCC | 50 |  |
| HCC1937 | ATCC | 23 |  |
| HCC202 | ATCC | 82 |  |
| HCC1599 | ATCC | 44 |  |
| HCC2218 | ATCC | 38 |  |
| HCC1428 | ATCC | 49 | Derived from MDA-MB-453;  Transfected with a mouse mammary tumour virus |
|  |  |  |  |
| MDA-kb2 | ATCC | 48 |  |
| Hs 605.T | ATCC | 61 |  |
| DU4475 | ATCC | 70 |  |
| Hs 606.T | ATCC | 44 |  |
| Hs 329.T | ATCC | 54 |  |
| Hs 371.T | ATCC | 28 |  |
| Hs 190.T | ATCC | 11 |  |
| Hs 344.T | ATCC | 49 |  |
| Hs 350.T | ATCC | 55 |  |
| Hs 849.T | ATCC | 41 |  |
| Hs 851.T | ATCC | 61 |  |
| Hs 875.T | ATCC | 21 |  |
| Hs 578Bst | ATCC | 74 |  |
| MCF 10A | ATCC | 36 | Derivative of MCF-10F;  Spontaneously immortalized |
|  |  |  |  |
| MCF-10-2A | ATCC | 36 | Derived from the same tissue  sample as MCF 10A and MCF 10F |
|  |  |  |  |
| MCF 10F | ATCC | 36 | Spontaneously immortalized |
| MCF-12A | ATCC | 60 | Spontaneously immortalized |
| MCF-12F | ATCC | 60 | Spontaneously immortalized |
| Hs 617.Mg | ATCC | 71 |  |
| HMT-3522 S1 | ECACC | 48 | Derived from HMT-3522 |
| HMT-3522 S2 | ECACC | 48 | Derived from HMT-3522 |
| HMT-3522 T4-2 | ECACC | 48 | Derived from HMT-3522 |
| MCF7/164R-4 | ECACC | 69 | Derived from MCF-7/S0.5 |
| MCF7/164R-7 | ECACC | 69 | Derived from MCF-7/S0.5 |
| MCF7/182R-6 | ECACC | 69 | Derived from MCF-7/S0.5 |
| MCF7/AnaR-1 | ECACC | 69 | Derived from MCF-7/S0.5 |
| MCF7/AnaR-3 | ECACC | 69 | Derived from MCF-7/S0.5 |
| MCF7/AnaR-4 | ECACC | 69 | Derived from MCF-7/S0.5 |
| MCF7 AREc32 | ECACC | 69 | Derived from MCF-7 |
| MCF7/ExeR-4 | ECACC | 69 | Derived from MCF-7/S0.5 |
| MCF7/LetR-1 | ECACC | 69 | Derived from MCF-7/S0.5 |
| MCF7/LetR-2 | ECACC | 69 | Derived from MCF-7/S0.5 |
| MCF7/LetR-3 | ECACC | 69 | Derived from MCF-7/S0.5 |
| MCF7/S0.5 | ECACC | 69 | Derived from MCF-7 |
| MCF7/TAMR-4 | ECACC | 69 | Derived from MCF-7/S0.5 |
| MCF7/TAMR-7 | ECACC | 69 | Derived from MCF-7/S0.5 |
| MCF7/TAMR-8 | ECACC | 69 | Derived from MCF-7/S0.5 |
| MCF7 AREc32 | ECACC | 69 | Derived from MCF-7 |
| MFM-223 | ECACC | >45 |  |
| T47D | ATCC, ECACC | 54 | Derived from T-47 |
| T47D-182R1 | ECACC | 54 | Derived from T47D/S5 |
| T47D-182R2 | ECACC | 54 | Derived from T47D/S6 |
| T47D/S2 | ECACC | 54 | Derived from T47D |
| T47D/S5 | ECACC | 54 | Derived from T47D |
| T47D/TR-1 | ECACC | 54 | Derived from T47D/S2 |
| ZR-75-1 | ECACC | 63 |  |
| UACC-893 | ATCC | 57 |  |
| UACC-3199 | ATCC | 58 |  |
| UACC-3133 | ATCC | 52 |  |
| UACC-732 | ATCC | 33 |  |
| AU-565 | ATCC | 43 |  |
| Other, N = 4 | | | |
| Hs 739.T | ATCC | 52 | Asian |
| HCC1954 | ATCC | 61 | East Indian |
| HCC1419 | ATCC | 42 | Hispanic |
| UACC-812 | ATCC | 43 | Mixed; Native American (50.48%) |
| Unspecified, N = 17 | | | |
| Hs 574.T | ATCC | Unspecified |  |
| Hs 566(B).T | ATCC | 35 |  |
| Hs 841.T | ATCC | 48 |  |
| Hs 861.T | ATCC | 42 |  |
| 184A1 | ATCC | 21 |  |
| 184B5 | ATCC | 21 |  |
| 1-7HB2 | ECACC | Unspecified | SV40 transformed cell line;  Derived from MTSV1-7 |
|  |  |  |  |
| fR2 | ECACC | Unspecified | SV40 transformed cell line |
| fR5 | ECACC | Unspecified | SV40 transformed cell line |
| SVCT | ECACC | Unspecified | SV40 transformed cell line |
| SVCT-MI2 | ECACC | Unspecified | SV40 transformed cell line;  Derived from SVCT |
|  |  |  |  |
| VP229 | ECACC | 47 |  |
| VP267 | ECACC | 48 |  |
| VP303 | ECACC | 69 |  |
| UACC-1179 | ATCC | 61 |  |
| UACC-2087 | ATCC | 51 |  |
| MTSV1-7 CE1 | ECACC | Unspecified | Derived from MTSV1-7 cell line;  Transfected with ERBB2 expression vector |
|  |  |  |  |

Data compiled from ATCC, ECACC, and the Swiss Institute of Bioinformatics

*Note.* Caucasian refers to WEA
